# Isolated Neutrophilic Urticarial Dermatosis in a Previously Healthy Male: A Case Report

**DOI:** 10.7759/cureus.59750

**Published:** 2024-05-06

**Authors:** Diala Alshiyab, Saleh A Ba-shammakh, Abdulqudos A Al-fakih

**Affiliations:** 1 Department of Dermatology, King Abdullah University Hospital, Irbid, JOR; 2 Faculty of Medicine, Jordan University of Science and Technology, Irbid, JOR; 3 Department of General Surgery, Ministry of Health, Amman, JOR; 4 Department of Public Health, Faculty of Medicine, Jordan University of Science and Technology, Irbid, JOR

**Keywords:** autoimmune disease, nds, nuds, neutrophilic dermatoses, neutrophilic urticarial dermatosis

## Abstract

Neutrophilic urticarial dermatosis (NUD), a variant falling under the larger umbrella of neutrophilic dermatoses (NDs), is characterized by distinctive clinical and histopathological attributes often associated with systemic conditions. This report presents a case of a 45-year-old male with no prior health issues who exhibits both clinical and pathological hallmarks of NUD without any concurrent systemic illness. This singular case illuminates the intricate aspects of NUD, emphasizing the necessity for accurate diagnostic methods and effective treatment strategies.

## Introduction

Neutrophilic dermatoses (NDs) comprise a group of disorders characterized by a significant presence of neutrophils within various skin layers, including the epidermis, dermis, and occasionally, the hypodermis. Conditions such as Sweet syndrome, pyoderma gangrenosum, and Sneddon-Wilkinson disease fall under the category of NDs [1-3]. The effort to consolidate these diverse conditions was spearheaded by Wallach and Vignon-Pennamen, underscoring the shared traits among these disorders [1-3]. These similarities include the non-infectious accumulation of neutrophils in a sterile environment, association with multiple systemic inflammatory and neoplastic conditions, involvement of various organ systems, and consistent responses to specific treatment regimens [1-3]. However, drawing distinct boundaries among the NDs proves challenging due to overlapping clinical and pathological features observable either in a single patient or across different disease stages [2,3]. These dermatoses are categorized into superficial, dermal, and deep types, each displaying unique manifestations, as demonstrated in conditions like pyoderma gangrenosum, Sweet syndrome, and Sneddon-Wilkinson disease [2,3]. Recent advancements in research underscore the pivotal role of neutrophils in the skin and potentially throughout the body, enhancing our understanding of NDs as predominantly autoinflammatory disorders originating from innate immune system dysfunctions [3-5].

Neutrophilic urticarial dermatosis (NUD) is a distinct type of ND, characterized by leukocytoclasia, interstitial and perivascular neutrophilic infiltration without dermal edema or vasculitis [5]. Although systemic symptoms such as fever or polyarthritis are commonly associated with NUD, there have been isolated cases showing significant markers of systemic inflammation without a specific underlying disease. The present case report explores an unusual occurrence of NUD in which the patient displays characteristic NUD features absent of prominent systemic manifestations.

## Case presentation

A 45-year-old male smoker with a known case of glucose-6-phosphate dehydrogenase deficiency (G6PDD) began experiencing various symptoms approximately two months before consulting at our outpatient clinic. Initially, he suffered from fever, night sweats, and upper respiratory issues, which were successfully managed with paracetamol, alleviating the fever. However, about 10 days after the initial feverish episode, he developed increasing low back pain and migratory arthralgia affecting several areas, including his proximal interphalangeal joints (PIPs), wrists, elbows, and knees. The pain was particularly pronounced during the early morning hours.

**Figure 1:**
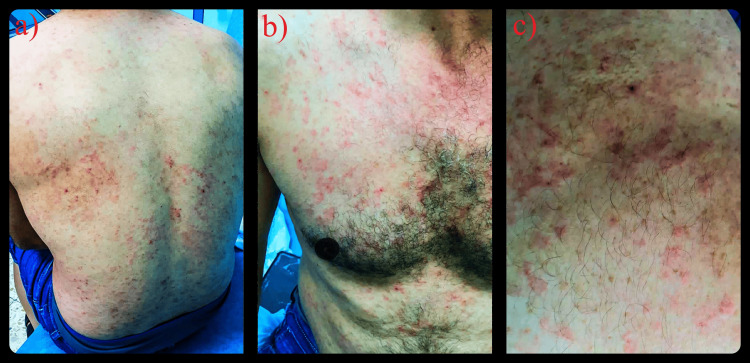
Initial Diffuse Erythematous Rash (a-c) Widespread, itchy erythematous rash observed on initial presentation. Images show affected areas on the back (a, c) and chest (b).

Over a two-month period, the patient lost 4 kg, reducing his weight from 86 kg to 82 kg. He also developed an erythematous, itchy skin rash that spread diffusely across his body (Figure 1). Concurrent with the skin symptoms, he experienced eye redness and itching, though ophthalmological examinations yielded non-significant findings. The patient had no previous similar symptoms, no history of atopy, and no family history of rheumatological disease or psoriasis.

The patient’s detailed history revealed that he had been suffering from low back pain for the past eight years, which seemed to improve with physical activity. Morning stiffness was brief, lasting only a few minutes, but occasionally intensified to a degree that disrupted his sleep. He also reported recurrent oral ulcers. However, there was no evidence of genital ulcers or Raynaud's phenomenon in his medical history.

Upon physical examination, his vital signs were normal, and a dermatological examination reported a diffuse erythematous rash. The eye examination confirmed the redness and itching he had been experiencing, and despite no observable swelling, the patient reported pain in the joints of his proximal interphalangeal joints (PIPs), wrists, elbows, and knees.

Laboratory investigations, including a complete blood count (CBC), kidney function test (KFT), liver function test (LFT), and urine analysis, were normal. Notably, tests for rheumatoid factor (RF), anti-SSB, anti-Sm, anti-Jo1, and anti-RNP, as well as hepatitis B and C, were negative. Radiological evaluations, including a CT scan, showed no abnormalities.

**Figure 2:**
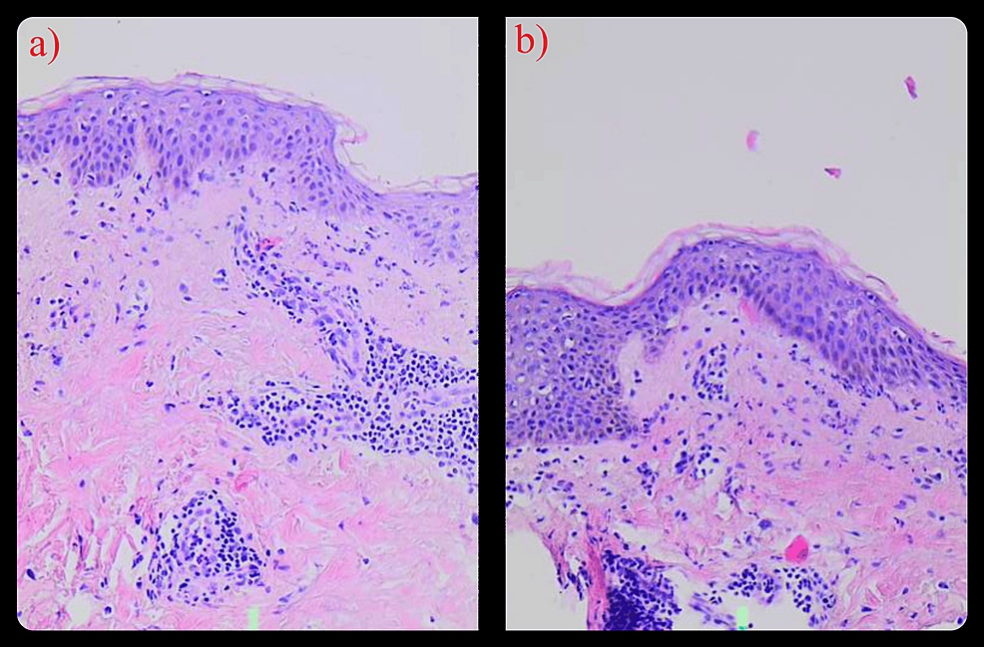
Histopathological Findings (a-b) Neutrophilic spongiosis, leukocytoclasia in the papillary dermis, scattered eosinophils, and peri-adnexal neutrophilic infiltrate. Chest (a); back (b).

In contrast, a histopathological examination of a skin biopsy provided more insight. The pathology report indicated neutrophilic spongiosis and the presence of neutrophils and leukocytoclasia in the papillary dermis, with scattered eosinophils. A peri-adnexal neutrophilic infiltrate was also noted, with intact blood vessel walls and no fibrinoid necrosis observed (Figure 2). These findings, along with his clinical presentation, confirmed the diagnosis of neutrophilic urticaria.

Given the diagnosis, a treatment plan was formulated. The patient was initially prescribed colchicine (0.5 mg every 12 hours) and prednisolone (10 mg) for management. One month later, the patient presented with a rash similar to the one initially depicted in Figure 1. According to the patient, there was no significant change in his condition throughout the month, and a repeat biopsy yielded findings identical to those shown in Figure 2. Due to his persistent symptoms and the limited therapeutic response, the colchicine dosage was increased to 0.5 mg every eight hours. Dapsone, which might have been the preferred therapeutic choice, was avoided due to the patient's G6PDD.

**Figure 3:**
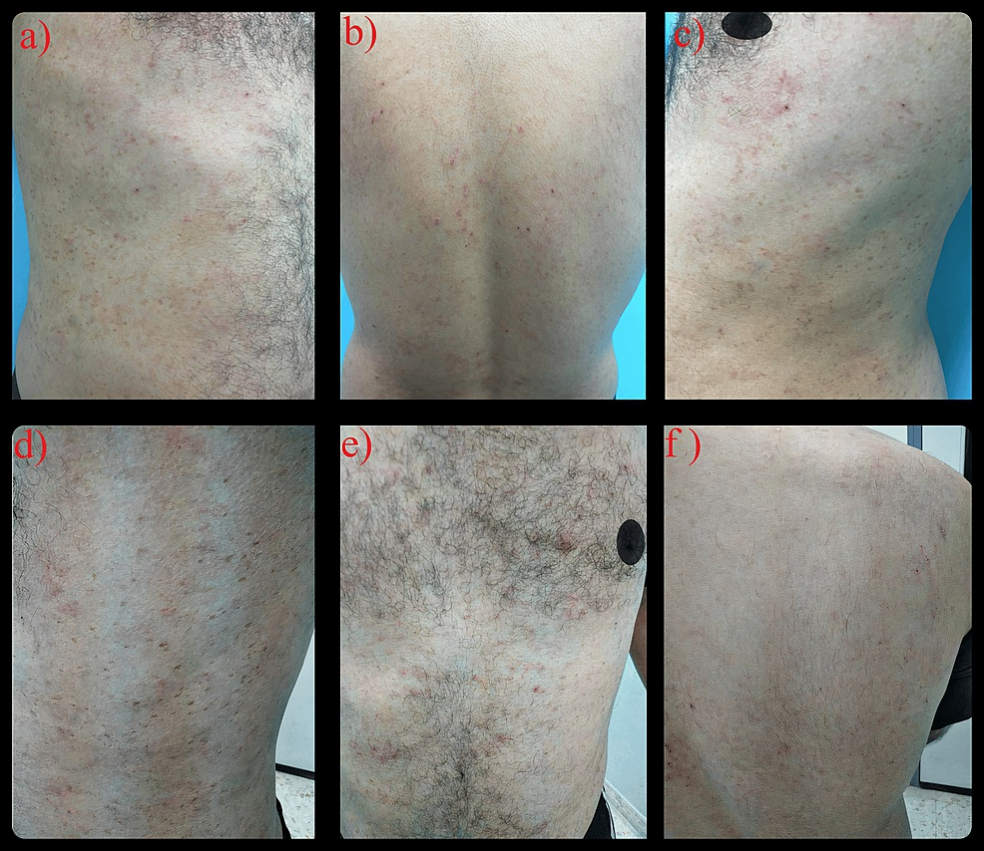
Skin Rash Improvement Post-Treatment Adjustment (a-f) Visible improvement in the erythematous rash is evident one week (a-c) and one month (d-f) after adjusting the colchicine treatment.

Follow-ups conducted at one week and one month showed significant improvement in the rash, as depicted in Figure 3. The same management plan was continued, with monthly follow-ups scheduled until the complete resolution of symptoms. The patient was instructed to attend all scheduled appointments and notify the clinic if the rash reappeared or worsened.

Considering the association of NUD with potential underlying disorders, the patient was advised to undergo regular check-ups every six months following the resolution of his rash.

## Discussion

NUD is distinguished by distinct clinical and histopathological characteristics within the group of neutrophilic dermatoses, predominantly associated with immune-mediated or autoinflammatory conditions [6]. Initially recognized as a distinct condition in 2009, knowledge and documentation of NUD have expanded, with over 120 documented cases showcasing a range of associated diseases [7]. Clinically, NUD is identifiable by pale or red patches, or minimally elevated plaques or papules, primarily appearing on the arms, torso, and legs, usually disappearing within 24 to 48 hours without leaving residual marks [7].

Accurate differentiation of NUD from other skin conditions, such as palisaded neutrophilic granulomatous dermatitis, Sweet syndrome, drug reactions, urticarial vasculitis, and typical urticaria, is vital due to its unique characteristics [6,7]. Unlike the severe itching typical of urticaria, NUD presents with dysesthesia and does not respond to antihistamines [3,7]. Histologically, neutrophilic epitheliotropism differentiates NUD from neutrophilic urticaria [3,7]. Additionally, the absence of eosinophils, papillary dermal edema, and specific alterations seen in drug eruptions and typical urticaria serve as significant indicators of NUD [7]. Despite the presence of leukocytoclasia, NUD differs from leukocytoclastic vasculitis as it does not exhibit noticeable vessel wall necrosis [3,6,7].

Systemic symptoms often accompany NUD, and their nature is usually determined by the associated disorder. These symptoms may include fever, joint pain, heightened inflammatory markers, and leukocytosis, with occasional reports of abdominal and chest discomfort [6,7]. Associations with adult-onset Still's disease, Schnitzler's syndrome, and systemic lupus erythematosus have been observed. Additionally, NUD may herald the onset of systemic juvenile idiopathic arthritis in children [8].

The pathophysiology of NUD is primarily attributed to abnormal activation of the innate immune response, involving inflammasome dysregulation and the IL-1 pathway [3,6,7]. Given its association with various diseases, multiple autoinflammatory pathways are likely involved [6,7].

Regarding management, treatment efficacy for NUD is variable. Traditional immunosuppressive therapies have had limited success, highlighting the need for personalized treatment approaches [6,7]. Medications inhibiting neutrophil migration, like colchicine and dapsone, show promise. Furthermore, IL-1 antagonists, including anakinra, have demonstrated potential, particularly in cases related to CAPS [3,6-10]. Rituximab has also been effective in treating resistant Schnitzler syndrome-associated NUD [10].

In conclusion, NUD is a unique clinical and histopathological entity within the spectrum of skin disorders. Understanding its systemic manifestations and underlying mechanisms is vital for effective management, as is recognizing its features and differentiating it from other dermatoses to devise successful treatment strategies.

## Conclusions

Although NUD is a recognized facet within the realm of NDs, it does not invariably coincide with systemic diseases, as illustrated in our study. Navigating the complexities of NUD requires careful differentiation from other potential diagnoses due to its varied clinical and histological manifestations. Addressing this condition therapeutically poses challenges due to the variability in patient responses. However, a deep understanding of the disorder, combined with the formulation of thoughtful treatment plans, offers hope for successful patient management. This case underscores the importance of continued research and refined insights into such rare occurrences in dermatological practice.
